# Thioredoxin Binding Protein-2 Regulates Autophagy of Human Lens Epithelial Cells under Oxidative Stress via Inhibition of Akt Phosphorylation

**DOI:** 10.1155/2016/4856431

**Published:** 2016-08-31

**Authors:** Jiaojie Zhou, Ke Yao, Yidong Zhang, Guangdi Chen, Kairan Lai, Houfa Yin, Yibo Yu

**Affiliations:** ^1^Eye Center, 2nd Affiliated Hospital, School of Medicine, Zhejiang University, Hangzhou, Zhejiang, China; ^2^Department of Otolaryngology, 2nd Affiliated Hospital, School of Medicine, Zhejiang University, Hangzhou, Zhejiang, China; ^3^Department of Public Health, School of Medicine, Zhejiang University, Hangzhou, Zhejiang, China

## Abstract

Oxidative stress plays an essential role in the development of age-related cataract. Thioredoxin binding protein-2 (TBP-2) is a negative regulator of thioredoxin (Trx), which deteriorates cellular antioxidant system. Our study focused on the autophagy-regulating effect of TBP-2 under oxidative stress in human lens epithelial cells (LECs). Human lens epithelial cells were used for cell culture and treatment. Lentiviral-based transfection system was used for overexpression of TBP-2. Cytotoxicity assay, western blot analysis, GFP/mCherry-fused LC3 plasmid, immunofluorescence, and transmission electronic microscopy were performed. The results showed that autophagic response of LECs with increased LC3-II, p62, and GFP/mCherry-LC3 puncta (*P* < 0.01) was induced by oxidative stress. Overexpression of TBP-2 further strengthens this response and worsens the cell viability (*P* < 0.01). Knockdown of TBP-2 attenuates the autophagic response and cell viability loss induced by oxidative stress. TBP-2 mainly regulates autophagy in the initiation stage, which is mTOR-independent and probably caused by the dephosphorylation of Akt under oxidative stress. These findings suggest a novel role of TBP-2 in human LECs under oxidative stress. Oxidative stress can cause cell injury and autophagy in LECs, and TBP-2 regulates this response. Hence, this study provides evidence regarding the role of TBP-2 in lens and the possible mechanism of cataract development.

## 1. Introduction

Age-related cataract is a multifactorial disease that plays a leading role in causing visual impairment (18.4%) and blindness (33.4%) in the world [1]. Although the molecular mechanisms of cataractogenesis still remain unclear, possible risk factors include ultraviolet rays, ionizing radiation, and chemical. All of these environmental factors generate reactive oxygen species (ROS) and disrupt the redox status in human lens epithelial cells (LECs) [2].

Thioredoxin (Trx) is a 12-KD protein which is ubiquitously expressed in all the living cells. Trx reduces a variety of substances to maintain the intracellular redox balance [3, 4]. Thioredoxin binding protein-2 (TBP-2), which is initially known as Vitamin D3 Upregulated Protein 1 (VDUP1) [5], can be regulated by mechanical stress, UV light, heat shock, hypoxia, H_2_O_2_, NO, glucose, and insulin [3]. TBP-2 is a negative regulator of the reducing capacities of Trx. TBP-2 binds with the reduced form of Trx and forms an intermolecular disulfide bond at the redox-active catalytic domain [6]. TBP-2 acts as a central regulator of cellular signaling pathways involved in the oxidative stress mechanism. It competes with peroxiredoxin (Prx) and apoptosis signal regulating kinase 1 (ASK1) to bind with Trx [7]. The binding of TBP-2 inactivates Trx, which breaks the Trx-ASK1 complex, allowing for the reactivation of ASK1 activity inducing apoptosis through activating JNK and p38 cascades [8]. Moreover, TBP-2 suppresses cellular proliferation along with cell cycle arrest and has been reported as a tumor suppressor gene [9, 10].

In human lens, previous studies showed that overexpression of TBP-2 increases cells' apoptosis under oxidative stress, which raises the question of whether TBP-2 might play an important role in cataract development [11]. Further investigation is needed to know the influence of TBP-2 on other aspects of lens cells and its potential therapeutic value in cataract.

Autophagy is a kind of cellular degradation pathway that maintains cellular homeostasis through degradation of intracellular proteins, lipids, and organelles in response to various environmental conditions [12]. Basal autophagy exists in all living cells at a relatively low level and is important for the prevention of aging [13]. Autophagy can be stimulated in numerous conditions, such as nutritional starvation, endoplasmic reticulum (ER) stress [14], increased reactive oxygen species (ROS) [15], reactive nitrogen species (RNS) [16], and senescence [17]. Depending on the circumstances, autophagy sometimes plays a protective role and sometimes accelerates cell damage and death. Autophagy has been classified into three types: macroautophagy (hereafter referred to as autophagy), microautophagy, and chaperone-mediated autophagy (CMA) [18]. The typical process of macroautophagy includes five stages: (1) initiation which depends on the cooperation of various autophagy-related proteins (Atg); (2) elongation that involves the extension of the LC3-tagged double-membrane phagophore; (3) maturation which is marked by the closure of autophagosomes; (4) fusion of the lysosomes; and (5) degradation of useless components for biomolecule recycling [12, 19, 20]. In mammals, the convergence of MAP1LC3/LC3 (microtubule-associated protein 1 light chain 3) from the free form LC3-I to the phosphatidylethanolamine-conjugated form (LC3-II) is a key step of autophagy. SQSTM1/p62 acts as an autophagic receptor. It binds to both ubiquitylated proteins via its ubiquitin associated (UBA) domain and LC3 via its LC3-interacting region (LIR). SQSTM1/p62 directs cargo to autophagosomes for degradation [21].

Mammalian target of Rapamycin (mTOR), which is a serine/threonine protein kinase, is an important regulator of stress-induced autophagy. Phosphatidylinositol 3-kinase- (PI3K-) Akt-mTOR signaling pathway is an antiapoptotic pathway which also plays an essential role in regulating macroautophagy. Akt could be activated through phosphorylation, and this process could be suppressed by wortmannin, 3-methyladenine (3-MA), and LY294002 [20, 22]. mTOR could be activated by PI3K/Akt via phosphorylation and then suppresses autophagy [23]. Recently, extensive studies illustrated that autophagy can be induced in mTOR-independent manner [20, 24–26].

Dysregulation of autophagy has been reported to be related to many diseases, such as Parkinson's disease, Alzheimer's disease, Crohn's disease, myopathy, and diabetes [27]. Currently, quite a few researches on autophagy in lens focus on the lens development and differentiation, which has elucidated that autophagy plays an essential role in the development of organelle-free zone (ORZ) [28, 29]. A few studies using genetically modified mouse models discussed the relationship between autophagy and the development of congenital cataract and age-related cataracts, but further studies are warranted to know the mechanisms [30–33].

Therefore, we studied the autophagic response of human lens epithelial cells induced by oxidative stress, which is regulated by TBP-2, to explore the possible mechanism of autophagy in the development of cataract.

## 2. Materials and Methods

### 2.1. Cell Culture and Treatment

Human lens epithelial cells (HLE-B3, also named LECs) (ATCC, USA), immortalized by infection with adenovirus 12-SV40, were grown and maintained in 1 : 1 Dulbecco's modified Eagle's medium and Ham's F-12 medium (DMEM/F-12) (#10-092-CV, Corning Cellgro, USA) which is supplemented with 10% fetal bovine serum (FBS) (Bioind, Israel), 100 U each of penicillin and streptomycin (Gibco, USA) in humidified CO_2_ incubator. The LECs, transfected by vector-control (vector) and TBP-2-overexpression lentivirus (TBP-2) were cultured in the same condition as original LECs. We used the cells within 8 passages for further experiments.

Cells were plated 24 hours before the experiments and experiments were performed when cell confluence reached 80%–90%. Before the treatment, cells were washed with PBS (137 mmol/L NaCl, 2.7 mmol/L KCl, 10 mmol/L Na_2_HPO_4_, 2 mmol/L KH_2_PO_4_, and pH 7.2–7.4) twice in order to avoid the influence of serum. Cells were treated with 50 *μ*M H_2_O_2_ in DMEM/F12 without serum for different durations.

For 3-MA treatment, 3-MA (1 mM) was pretreated for 3 hours and then used in combination with H_2_O_2_ (50 *μ*M) for 12 hours. For wortmannin treatment, H_2_O_2_ (50 *μ*M) in serum-free medium was treated for 12 hours. 10 *μ*M wortmannin (#S2758, Selleck, USA) was added to the medium six hours prior to the end of the treatment. For bafilomycin A1 treatment, 50 nM bafilomycin A1 (#BF1116, Sangon, China) was added to the medium 4 hours before the end of the 12-hour H_2_O_2_ treatment.

### 2.2. Construction of Lentiviral-Based TBP-2-Overexpressing LECs

The mammalian expression vector LV4 which is used for overexpression was obtained from Shanghai GenePharma. Primers were designed according to the sense cDNA of TBP-2. The forward primer was TBP-2-F: CGGCGGCCGCATGGTGATGTTCAAGAAGAT, while the reverse primer was TBP-2-R: CGATGCATTCACTGCACATTGTTGTTGA. With high-fidelity DNA polymerase (Phusion, Thermo Fisher), the sequence of TBP-2 with NotI and NsiI at both ends was amplified and introduced to the multiple cloning sites of LV4 vector. Then we used the LV4-TBP-2 which had been sequenced exactly to cotransfect Human Embryonic Kidney (HEK293T) cells with 3 other packaging plasmids (pGag/Pol, pRev, and pVSV-G) to produce lentiviral particles. Forty-eight hours after transfection, the supernatant was collected and filtered with a 0.45 *μ*m membrane (Millipore) and then condensed with ultracentrifugation. The lentivirus particles were stored at −80°C. We calculated lentivirus titer by testing the transfection efficiency in HEK293T cells. Finally, we transfected LECs with the lentivirus and monitored the percentage of cells, which were tagged with green fluorescent protein (GFP) under fluorescent microscopy. The multiplicity of infection (MOI) we used on LECs was 5. Overexpression of TBP-2 in LECs was confirmed by western blot.

### 2.3. RNA Interference

RNA interference was achieved by transfection of siRNA by Lipofectamine 2000. The siRNAs were designed and synthesized by Shanghai GenePharma. The sequence of siRNA specific against TBP-2 (si-TBP-2) was sense 5′-GAGACCUGGAAACAAAUAUTT-3′ and antisense 5′-AUAUUUGUUUCCAGGUCUCTT-3′. The sequence of siRNA specific against Atg5 (si-Atg5) was sense 5′-GACGTTGGTAACTGACAAATT-3′ and antisense 5′-UUUGUCAGUUACCAACGUCTT-3′. The sequence of scramble siRNA for negative control (NC) was sense 5′-UUCUCCGAACGUGUCACGUTT-3′ and antisense 5′-ACGUGACACGUUCGGAGAATT-3′. Experiments were conducted according to the manufacturer's protocols. Forty-eight hours after transfection, subsequent experiments were performed.

### 2.4. Cytotoxicity Assay

Cytotoxicity assays were performed using a Cell Counting Kit-8 (CCK-8) assay (Dojindo, Japan). Twenty-four hours before the treatment, 1 × 10^4^ cells were seeded in each well of the 96-well plate. Then, the cells were rinsed with PBS twice and incubated with indicated concentrations of H_2_O_2_ dissolved in DMEM/F12 without serum for 24 hours. The control group, which was defined as no viability loss, was cultured under normal conditions without H_2_O_2_. After the treatment, the medium was removed and the cells were washed with PBS. Then, each well was refilled with 90 *μ*L of DMEM/F12 supplemented with 10% fetal bovine serum and 10 *μ*L of CCK-8 reagents and incubated in 37°C for 3 hours. The cell viability was evaluated with OD450 values using a 96-well microplate reader (Bio-Rad, USA).

### 2.5. Western Blot Analysis

After the indicated treatment, cells were lysed and the BCA Protein Assay Kit (Sigma, B9643-1L) was used to determine the total protein concentration. Same amounts of protein were subjected to SDS-PAGE with certain concentrations, transferred to a polyvinylidenedifluoride (PVDF) membrane (Millipore, USA) and blocked for 1 hour with 5% nonfat dry milk in Tris buffered saline (TBS-T, 10 mM Tris-HCl [pH 7.5], 100 mM NaCl, and 0.1% Tween 20). Primary antibody incubations were performed at the following dilutions: TBP-2, 1 : 200 (#K0204-3, MBL, USA); LC3B, 1 : 1000 (#L7543, Sigma-Aldrich, USA); P62, 1 : 1000 (#sc-28359, Santa Cruz Biotech, USA); Atg5, 1 : 1000 (#9980, CST, USA); mTOR, 1 : 1000 (#2972, CST, USA); Phospho-mTOR (Ser2448), 1 : 1000 (#2971, CST, USA); GAPDH, 1 : 5000 (#M20006, Abmart, China); Akt (pan), 1 : 1000 (#4691, CST, USA); Phospho-Akt (Ser473), 1 : 2000 (#4060, CST, USA); and *β*-actin, 1 : 10000 (#A5441, Sigma-Aldrich, USA). All incubations were done at 4°C overnight. Then, the membrane was washed thrice with TBS-T for 30 minutes and followed by respective secondary antibody incubations with goat anti-rabbit/mouse IgG-horseradish peroxidase (Santa Cruz, USA). Immunodetection was performed with the ECL Western Blot Detection System (Millipore, USA). The immunoblot was analyzed with an imaging system (ChemiDoc*™* MP System, Bio-Rad, USA). The intensities of the bands were quantified by densitometry using ImageJ (NIH, USA). Values were reported as percentage of the control values ± SEM.

### 2.6. GFP-LC3 and mCherry-LC3 Fluorescence Microscopy

The GFP-LC3 and mCherry-LC3 plasmids which encode fusion proteins of fluorescent protein and LC3 were generously gifted by Professor Chen GD. GFP-LC3 was used in wildtype LECs. mCherry-LC3 was used in the LECs transfected with lentiviral plasmids in order to avoid overlapping of green fluorescence.

Lipofectamine 2000 was used for transfection of the plasmids according to the manufacturer's standard protocol (Invitrogen, USA). Twenty-four hours after transfection, cells were washed with PBS thrice and then treated with complete medium (control), serum-free medium with 50 *μ*M H_2_O_2_ (H_2_O_2_), or serum-free medium with 10 nM Rapamycin (Rapamycin) for 24 hours. In the wortmannin and bafilomycin A1 inhibitory experiments, cells were treated with the control groups serum-free medium containing 50 *μ*M H_2_O_2_ (control) or 50 *μ*M H_2_O_2_ plus 10 *μ*M wortmannin (Wortmannin) or plus 100 nM bafilomycin A1 (Bafilomycin A1) for 24 hours.

After the indicated treatments, cells were fixed with 4% paraformaldehyde (Sangon, China). Nuclei were stained with 1 *μ*g/mL DAPI (D9564, Sigma, USA). Coverslips were mounted onto microscope slides using fluorescence mounting medium (H-1000, Vector, USA).

Microscopic study was performed on Olympus IX71 Inverted Biological Microscope and Olympus BX61W1-FV1000 confocal laser scanning microscope.

### 2.7. Immunofluorescence Staining

Cells were seeded on cleaned coverslips 24 hours prior to the treatment. After H_2_O_2_ exposure, the cells were fixed in 4% formaldehyde solution. Next, the cells were permeabilized with 0.2% Triton-X-100 (Sigma-Aldrich, USA) and blocked with 20% donkey serum (Bioind, Israel). Subsequently, the cells were incubated with anti-LC3B (L7543, Sigma-Aldrich, USA) overnight at 4°C. After thorough washing with PBS, Alexa Fluor® 555 Donkey Anti-Rabbit IgG (Heavy+Light chains) (A-31572, Invitrogen, USA) solution was used to incubate the cells for 1.5 hours at room temperature. The nuclei were stained with DAPI. Eventually, the coverslips were mounted on the slides with a drop of mounting medium (H-1000, Vector, USA). Fluorescent images were captured with a Zeiss LSM laser scanning confocal microscope.

### 2.8. Transmission Electron Microscopy

The cells were fixed with 7% glutaraldehyde. Paraffin embedding and dehydration were performed subsequently. Ultrathin sections were stained with uranyl acetate and lead citrate. Images were taken with JEM-1200EX Transmission Electron Microscope at 120 V.

### 2.9. Statistics

All data were analyzed using two-tailed Student's *t*-test. Statistical significance was considered as *P* value <0.05. Error bars represent standard errors.

## 3. Results

### 3.1. Low Concentration of Oxidative Stress Triggers Autophagy in LECs

**Figure 1:**
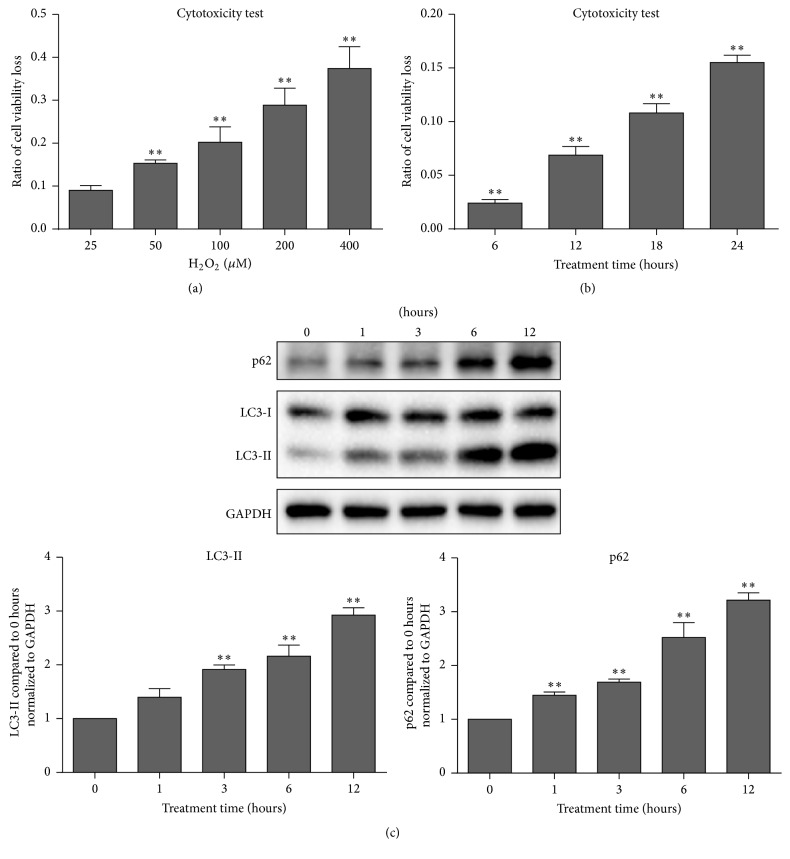
Oxidative stress decreased cell viability and triggered autophagy. (a) Twenty-four hours after the indicated dose of H_2_O_2_ treatment, CCK-8 assay revealed that cell viability loss was in a dose-dependent manner. (b) When cells were treated with 50 *μ*M H_2_O_2_ for various time durations, cell viability loss presented a time-dependent manner. (c) With the time increased, oxidative treatment with 50 *μ*M H_2_O_2_ induced LC3-II and p62 expression significantly. Data were presented as mean ± SEM of three independent experiments. ^*∗∗*^ *P* < 0.01.

Firstly, a series of preexperiments were performed to verify that oxidative stress causes cell injury depending on time and concentration. When LECs were treated with various concentrations of H_2_O_2_ for 24 hours, cell viability loss was increased significantly (*P* < 0.01) (Figure 1(a)). When LECs were treated with 50 *μ*M H_2_O_2_ for various time periods, cell viability loss was increased in a stepwise manner significantly (*P* < 0.01) (Figure 1(b)). Western Blot assays demonstrated a time-dependent induction of LC3-II and p62 (Figure 1(c)).

**Figure 2:**
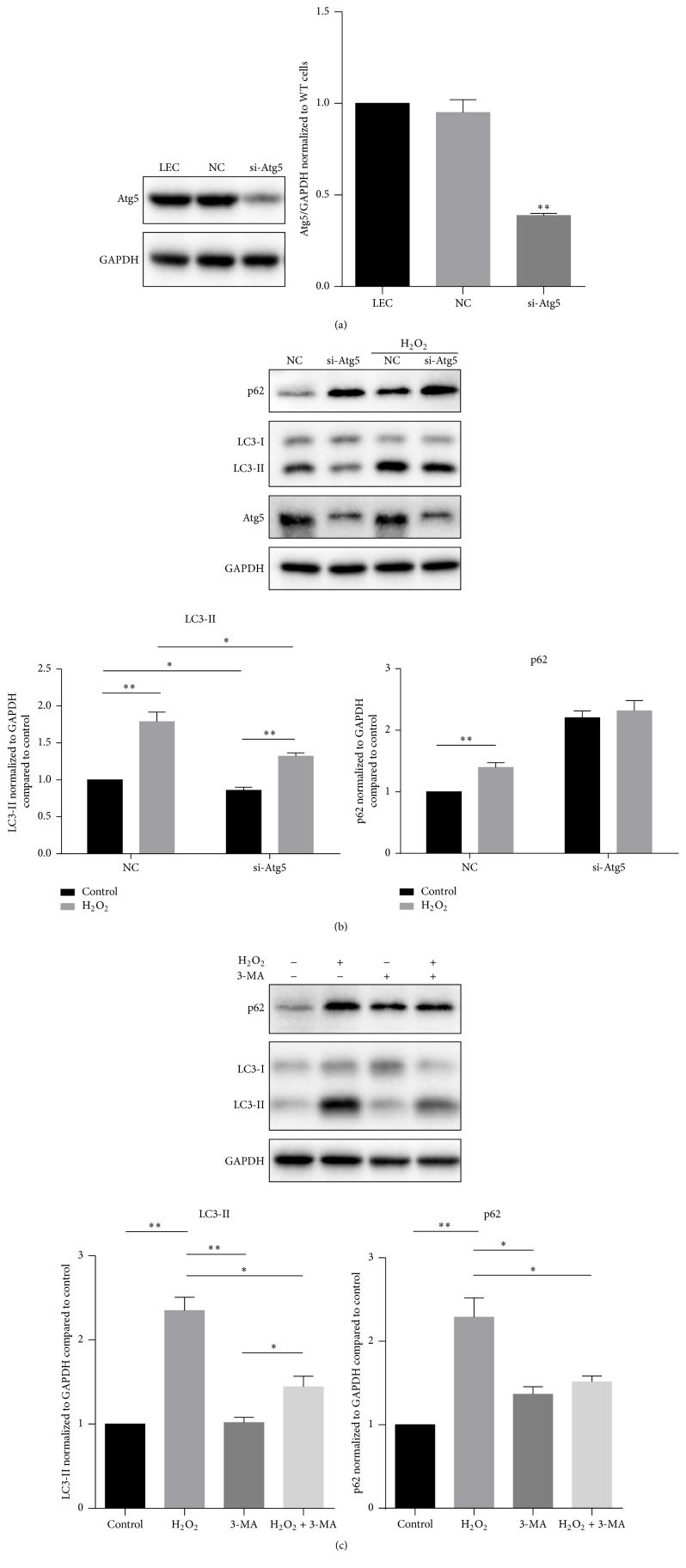
The upregulation of p62 is a unique response in LECs during autophagy induction. (a) Knockdown of Atg5 successfully suppressed Atg5 expression to nearly 38%. (b) Knockdown of Atg5, which suppressed autophagy initiation, prevented increase of p62 accumulation after H_2_O_2_ (50 *μ*M) treatment for 12 hours. (c) Suppression of autophagy initiation with 3-MA also decreased p62 accumulation after H_2_O_2_ (50 *μ*M) treatment for 12 hours. These findings demonstrated that p62 increased with autophagy induction. Data were presented as mean ± SEM of three independent experiments. ^*∗*^ *P* < 0.05. ^*∗∗*^ *P* < 0.01.

To figure out whether it is autophagic defect that caused accumulation of p62, we firstly blocked autophagy at an early stage through knockdown of* Atg5* expression, which is essential for Atg5-Atg12-Atg16L complex formation [12].* Atg5* specific siRNA (si-Atg5) successfully suppressed* Atg5* to nearly 38% of the control (*P* < 0.01) (Figure 2(a)). Knockdown of* Atg5* significantly suppressed both basal autophagy under normal conditions (*P* < 0.05) and inductive autophagy under oxidative stress (*P* < 0.05), as indicated by decreased expression level of LC3-II. When the* Atg5* was knocked down, p62 expression level showed no significant difference between the control and oxidative treatment groups (Figure 2(b)). These findings indicated that the increase of p62 was not due to autophagic defect. To confirm this, we treated the cells with 3-MA, a phosphoinositide 3-kinase (PtdIns3K) inhibitor that effectively blocks the early stage of autophagy [22]. The addition of 3-MA decreased both LC3-II and p62 expression level in comparison with single H_2_O_2_ treatment (Figure 2(c)). These data demonstrated that the elevation of p62 was a unique response in LECs when autophagy was induced under oxidative stress.

**Figure 3:**
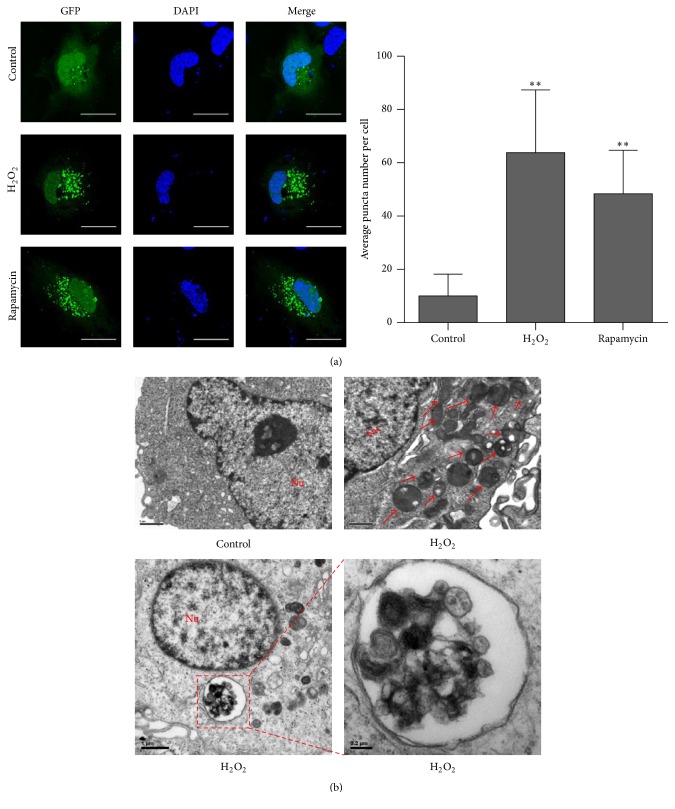
Autophagosomes induced by oxidative stress were observed with fluorescent and transmission electronic microscopy (TEM). (a) Twenty-four hours after transfection with GFP-LC3 plasmid, LECs were then cultured with normal medium (control), 50 *μ*M H_2_O_2_ (H_2_O_2_), or 10 nM Rapamycin (Rapamycin) for 24 hours. 10 nM chloroquine was cotreated at the same time. After fixation, GFP fluorescence was observed with confocal microscopy. The average number of GFP dots per cell was significantly increased in H_2_O_2_ group and Rapamycin group (*P* < 0.01). Scale bar, 25 nm. (b) TEM analyses showed a great number of single/double-membrane vesicles in H_2_O_2_ treated cells. Data were presented as mean ± SEM of three independent experiments. ^*∗∗*^ *P* < 0.01. The lower right image, scale bar 0.2 *μ*m. The rest of the images, scale bar 1 *μ*m.

The GFP-LC3 puncta assay can evaluate the autophagic response of LECs under oxidative stress. When cells were cultured with chloroquine (10 nM) in normal medium, the green fluorescence was distributed both in the nucleus and in cytoplasm and it was more condensed in the nucleus. We could see just a few dots in the cytoplasm (average number of dots per cell = 10.00 ± 1.496, *N* = 30). The positive control, which was treated by Rapamycin (10 *μ*M) and chloroquine (10 nM) in DMEM/F12 without serum for 12 hours, showed aggregated green fluorescent puncta in the cytoplasm, and the green fluorescence in the nucleus decreased. The similar phenomena were found when cells were treated with H_2_O_2_ (50 *μ*M) and chloroquine (10 nM) in serum-free medium; the average number of puncta per cell dramatically increased (63.83 ± 4.292, *N* = 30). These data indicated that autophagosomes, which were marked by GFP-conjugated LC3, were formed in the cytoplasm (Figure 3(a)).

The transmission electron microscopy (TEM) analyses showed a great number of single/double-membrane vesicles in H_2_O_2_ treated cells (Figure 3(b)).

In all, low dose of oxidative stress induced autophagic response in LECs.

### 3.2. Overexpression of TBP-2 Promotes Autophagy in LECs under Oxidative Stress

**Figure 4:**
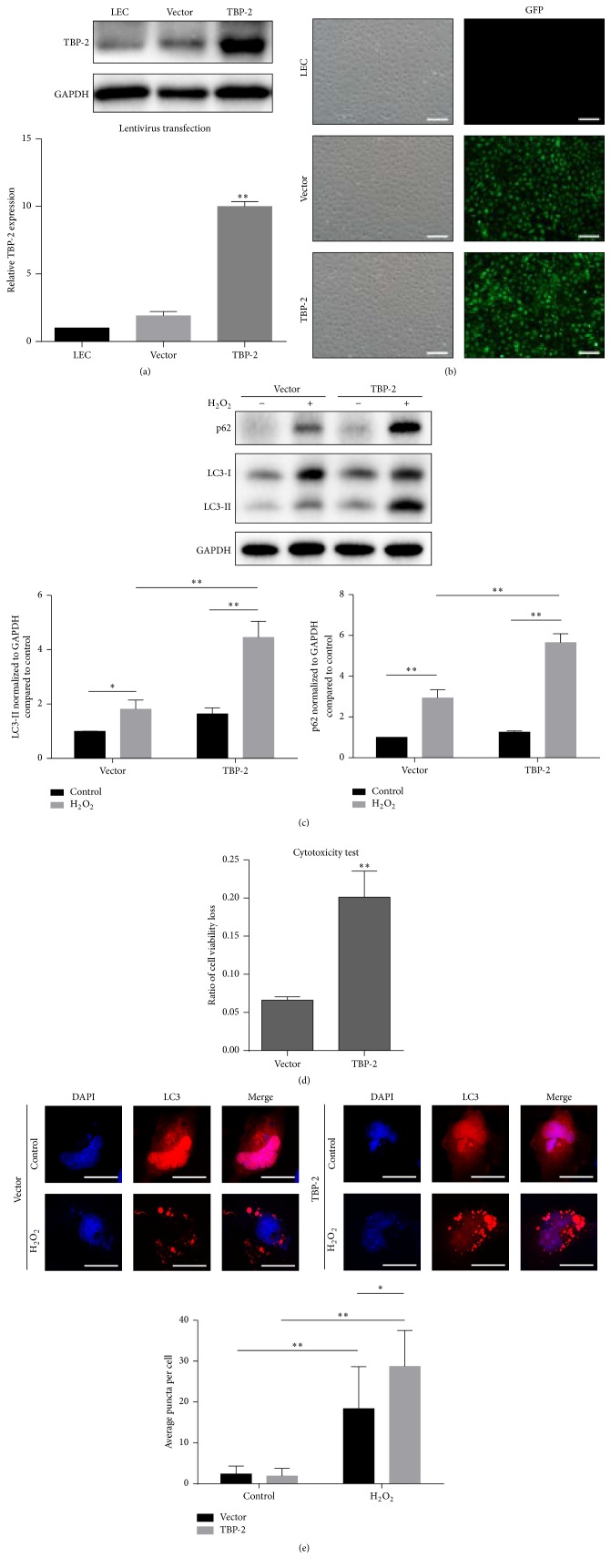
Overexpression of TBP-2 promoted cell injury and autophagy in LECs under oxidative stress. (a-b) Lentiviral-based TBP-2-overexpressing showed a nearly 10-fold increase of TBP-2 level. Fluorescent microscopy showed a high transfection efficiency. Scale bar, 200 nm. (c) After treatment with H_2_O_2_ (50 *μ*M) for 12 hours, the LECs overexpressing TBP-2 (TBP-2) showed a higher level of LC3-II and p62 than the ones transfected with empty vector (vector). (d) Overexpression of TBP-2 worsened cell viability under oxidative stress. (e) Twenty-four hours after transfection with mCherry-LC3 plasmid, cells were then cultured with normal medium (control) or 50 *μ*M H_2_O_2_ (H_2_O_2_) for 24 hours. mCherry-LC3 puncta assay revealed a larger average number of mCherry dots per cell in TBP-2-overexpressing LECs under oxidative stress. Scale bar, 25 nm. Data were presented as mean ± SEM of three independent experiments. ^*∗*^ *P* < 0.05, ^*∗∗*^ *P* < 0.01.

To investigate the influence of overexpression of TBP-2 on autophagy in LECs under oxidative stress, we firstly established TBP-2 stable overexpression cells (Figures 4(a)-4(b)). Then the cells were treated with 50 *μ*M H_2_O_2_ in DMEM/F12 without serum for 12 hours. The results showed that the LC3-II and p62 expression level of TBP-2 cells were significantly elevated, indicating that, with the overexpression of TBP-2, LECs are more likely to respond to oxidative stress with induced autophagy (Figure 4(c)).

Next, we evaluated the cell viability and found that the cell viability loss significantly increased in TBP-2 when compared to that of vector group (*P* < 0.01) (Figure 4(d)). mCherry-LC3 puncta assay showed that TBP-2 (28.73 ± 1.606,  *N* = 30) had significantly more mCherry-positive spots than that of vector group (18.40 ± 1.870,  *N* = 30), which indicated higher level of autophagic flux when treated with H_2_O_2_ (*P* < 0.05) (Figure 4(e)). Meanwhile, we have also found that a larger number of micronuclei existed in the cells.

These findings revealed that overexpression of TBP-2 promoted the autophagic response of LECs under oxidative stress, with higher cell viability loss, which indicates that LECs with overexpression of TBP-2 would be more sensitive to oxidative stress.

### 3.3. Knockdown of TBP-2 Decreases Level of Autophagic Flux under Oxidative Stress

**Figure 5:**
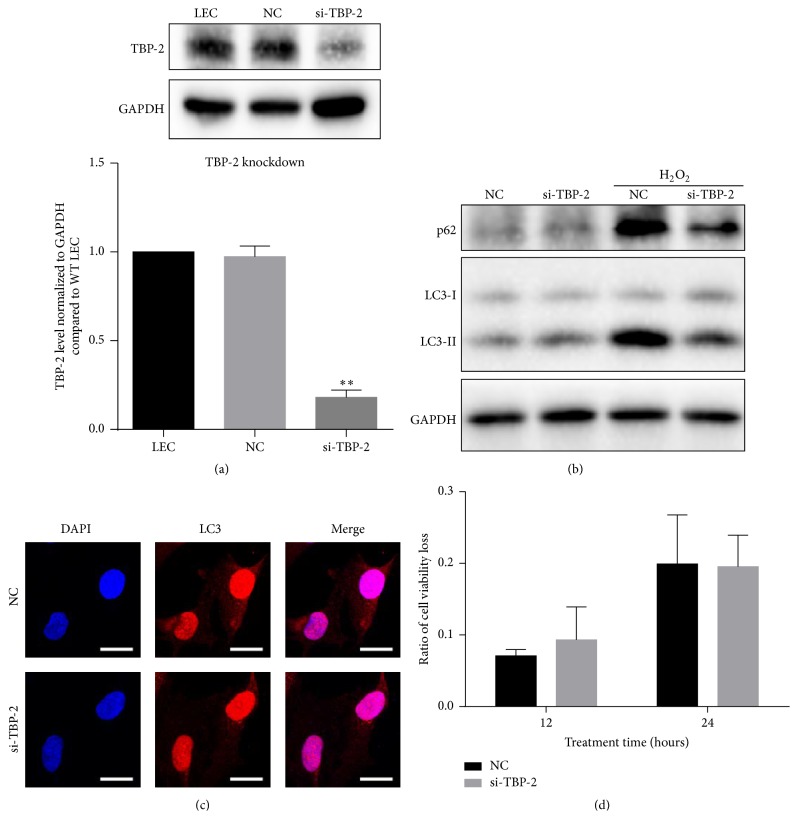
Knockdown of TBP-2 attenuated the autophagic response in LECs under oxidative stress. (a) Knockdown of TBP-2 successfully inhibited TBP-2 expression to about 18%. (b) Twenty-four hours after transfection, cells were treated with H_2_O_2_ (50 *μ*M) for 12 hours. Western blot showed that si-TBP-2 cells had lower LC3-II and p62 level than NC cells. (c) Immunofluorescent detection of LC3 indicated lower intensity in the cytoplasm of si-TBP-2 cells than NC cells. (d) Cytotoxicity tests revealed that, after 12 and 24 hours of H_2_O_2_ treatments, no significant difference was found in si-TBP-2 cells and NC cells. Scale bar, 25 nm. ^*∗∗*^ *P* < 0.01.

To confirm the autophagy promoting effect of TBP-2, we next performed the loss-of-function study. Forty-eight hours after transfection, the quantity of TBP-2 in the si-TBP-2 cells dropped to 18% of that in the control cells (Figure 5(a)).

We found that, after 12 hours of oxidative treatment, both the LC3-II and p62 expression levels of si-TBP-2 were significantly lower than NC (Figure 5(b)). These findings apparently revealed that the knockdown of TBP-2 inhibited the autophagic response in LECs under oxidative stress. Immunofluorescent staining of LC3 showed relatively higher intensity in NC after 12 hours of H_2_O_2_ treatment, which agreed with the immunoblot result (Figure 5(c)). Cytotoxicity tests showed no significant differences between NC and si-TBP-2 after 12 and 24 hours of H_2_O_2_ treatment (Figure 5(d)).

Therefore, knockdown of TBP-2 suppressed the autophagic response of LECs. It showed tiny effect on cell viability loss under oxidative stress.

### 3.4. TBP-2 Regulates Autophagy Mostly at the Initiation Stage of Autophagic Flux

To further determine the specific stage of autophagy that TBP-2 may regulate, we used wortmannin to inhibit autophagy at the initiation stage and bafilomycin A1 to block the fusion of autophagosomes and lysosomes [22].

**Figure 6:**
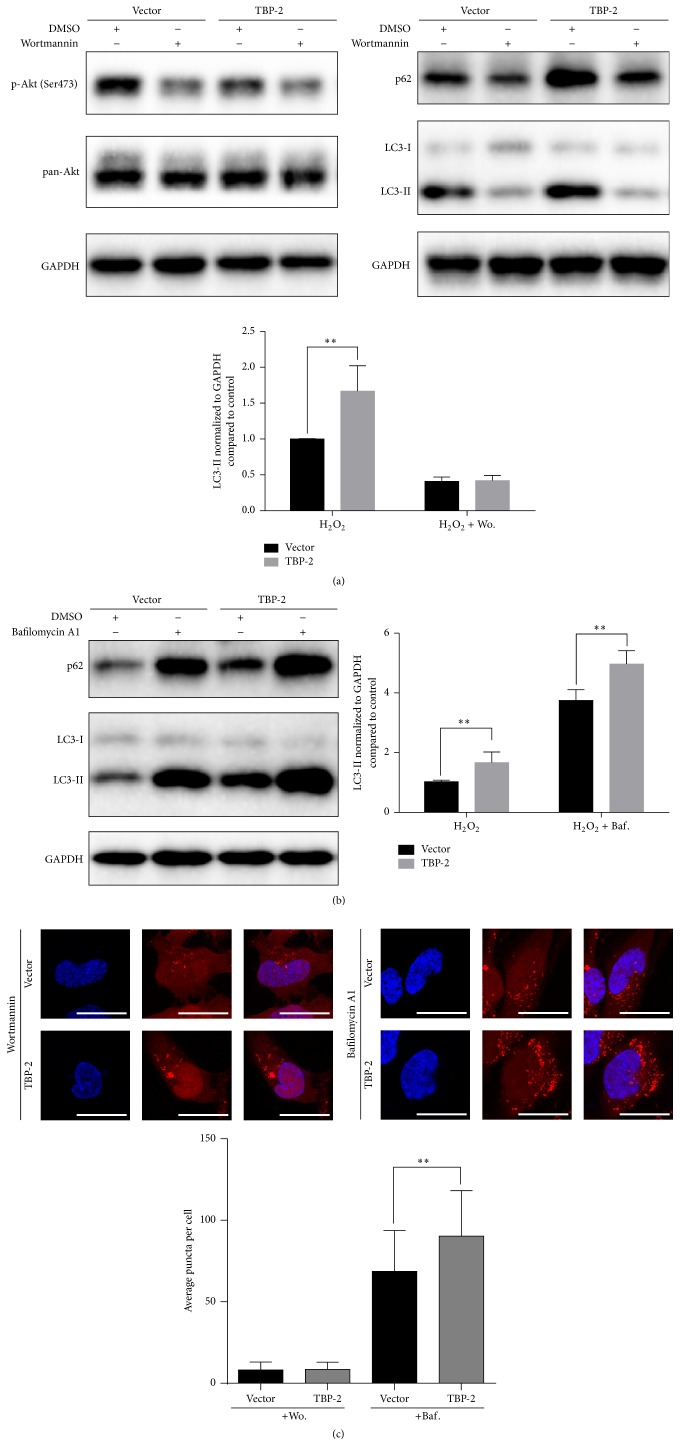
TBP-2 mostly regulates autophagy in the initiation stage. (a) Vector cells and TBP-2 cells were treated with H_2_O_2_ (50 *μ*M) for 12 hours, and wortmannin (10 *μ*M) was added in the last 6 hours. The addition of wortmannin treatment decreased the phosphorylation of Akt at Ser473. The expression level of LC3-II in both types of cells decreased. (b) When bafilomycin A1 was added in the last 4 hours of the same H_2_O_2_ treatment, TBP-2 cells showed significantly higher level of LC3-II than NC cells (*P* < 0.01). (c) mCherry-LC3 puncta assay of the previous treatments showed similarly small number of LC3 dots in both cells when treated with 3-MA, and TBP-2 cells had significantly larger number of LC3 dots than NC cells when treated with bafilomycin A1 (*P* < 0.01). Data were presented as mean ± SEM of three independent experiments. ^*∗∗*^ *P* < 0.01. Scale bar, 25 nm.

We found that when wortmannin was used, the phosphorylation of Akt at Ser473 was inhibited. The expression of LC3-II and p62 of both vector and TBP-2 dropped, without significant difference between them (Figure 6(a)). mCherry-LC3 puncta assay showed that, after wortmannin treatment, the red fluorescence distributed diffusely in the cytoplasm, with relatively higher intensity in the nuclei. There was no significant difference in the average number of puncta per cell between vector and TBP-2 (Figure 6(c)). These findings indicated that the autophagic flux of both TBP-2 and vector-control was suppressed by wortmannin at the initiation stage.

When bafilomycin A1 was used, we found that the expression of LC3-II and p62 of TBP-2 was significantly higher than that of the vector (*P* = 0.0004) (Figure 6(b)). mCherry-LC3 puncta assay exhibited that, after bafilomycin A1 treatment, the red fluorescence accumulated in the cytoplasm, with less intensity in the nuclei, which represented undergraded autophagosomes. The number of puncta per cell was significantly higher in TBP-2 compared to vector (Figure 6(c)). These findings revealed that TBP-2 induces more autophagic flux.

Therefore, TBP-2 mainly regulates autophagy in the initiation stage and facilitates autophagic induction.

### 3.5. The Influence of TBP-2 on Autophagy Is Independent of the Phosphorylation of mTOR but Regulated by Akt

**Figure 7:**
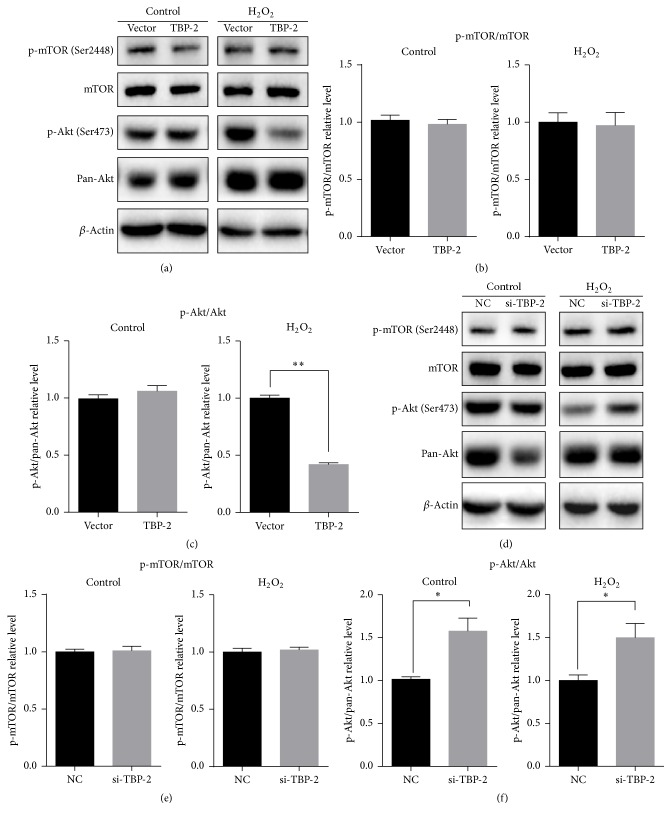
TBP-2's influence on autophagy is independent of the phosphorylation of mTOR but regulated by Akt. (a–c) Overexpression of TBP-2 inhibited Akt phosphorylation at Ser473 under oxidative stress (*P* < 0.01). Phosphorylation of mTOR at Ser2448 showed no difference. (d–f) Knockdown of TBP-2 increased Akt phosphorylation rate (*P* < 0.01). Phosphorylation of mTOR at Ser2448 also showed no difference. Data were presented as mean ± SEM of three independent experiments. ^*∗*^ *P* < 0.05. ^*∗∗*^ *P* < 0.01.

As shown in Figure 7, whether if treated with H_2_O_2_ or not, the expression of mTOR presented no significant difference. The phosphorylation of mTOR at Ser2448, which is a direct phosphorylation site of Akt, has no significant difference (Figures 7(a) and 7(b)). Similar results were obtained when* TBP-2* was knocked down (Figures 7(d) and 7(e)). The phosphorylation of mTOR is a key step of PI3K/Akt/mTOR pathway. Our study revealed that TBP-2's impact on LECs under oxidative stress is independent of the activation of mTOR by Akt.

On the other hand, the phosphorylation of Akt is highly related to TBP-2. When TBP-2 was overexpressed, the phosphorylation ratio of Akt at Ser473 showed no significant difference in normal conditions and dropped significantly under oxidative stress conditions (*P* < 0.01) (Figures 7(a) and 7(c)). Knockdown of* TBP-2* made the phosphorylation ratio of Akt stably higher than NC no matter if treated with H_2_O_2_ or not (*P* < 0.05) (Figures 7(d) and 7(f)). Through these approaches, we have illustrated that TBP-2 downregulates the phosphorylation of Akt at Ser473 in LECs under oxidative stress. Combining with the data in Figure 6(a) which illustrated the autophagy-regulating effect of TBP-2 was suppressed when Akt was inactivated by wortmannin, we were convinced that TBP-2 mostly regulates autophagy through Akt.

These data suggested that TBP-2 regulates autophagy in LECs under oxidative stress through activation of PI3K/Akt pathway but not the activation of mTOR.

## 4. Discussion

As the leading cause of visual impairment and blindness in the world [1], cataract, especially its possible etiology and pathogenesis, attracts people's attention all the time. We have demonstrated that autophagy was induced in LECs under oxidative stress. Then, we used overexpression and knockdown of* TBP-2* to illustrate its functional characteristics. TBP-2, which is widely accepted throughout the world as a negative regulator of Trx's antioxidative functions, regulates autophagy induced by oxidative stress. The most possible autophagic stage that TBP-2 regulates is the initiation stage, and we have found that the inhibition of Akt phosphorylation caused by TBP-2 contributes to it.

### 4.1. Autophagy Was Triggered by ROS in LECs

Numerous studies have elucidated the essential role of ROS in the induction of autophagy. Excessive accumulation of ROS breaks intracellular homeostasis, and autophagy in turn reduces oxidative impairment by engulfing and degrading irreversibly oxidized substances [34]. In the field of cataract research, convincing data of the relation between oxidative stress and autophagy in human lens are still lacking. In our study, we first found that the oxidative damage caused by H_2_O_2_ is time- and dose-dependent. Then western blot, GFP-puncta assay, and TEM which are general methods for monitoring autophagy were used and verified the autophagic response.

Interestingly, the expression level of p62 was increased after the treatment with H_2_O_2_. This phenomenon goes against our common notion that p62 degradation represents increased autophagic flux. p62 changes could be cell type and context specific [22]. p62 is an important regulator of Nrf2/Keap1 antioxidant signaling pathway; there is a positive feedback loop between Nrf2 and p62 as a response to oxidative stress [35]. The increasing quantity of p62 can be diminished by autophagy, but excessive p62 as a consequence of insufficient autophagy can accumulate and harm the intracellular homeostasis. However, sometimes the increasing p62 is merely a consequence of transcriptional upregulation. Previous studies have reported that eIF2*α*/ATF4 pathway and RAF1/Raf-MAP2K/MEK-MAPK/ERK pathway upregulate p62 expression during autophagy [36, 37]. To better understand, we suppressed autophagy initiation with* Atg5*-knockdown and 3-MA. We have found that no matter if with or without H_2_O_2_ treatment, knockdown of Atg5 stabilized* p62* expression. If it was autophagic defect, dramatic increase of p62 should have been observed when autophagy was inhibited in the early stage. This standpoint was also proved by inhibiting autophagy initiation with 3-MA. Thus, the transcriptional upregulation of p62 is highly suspected. Therefore, the complexity of p62 level should not be ignored when we use this marker to interpret autophagy. Further exploration regarding the activation of related signaling pathway can be considered in the future research.

### 4.2. The Regulating Effect of TBP-2 on Autophagy Induced by ROS

Apart from the negative regulation of antioxidative function of TBP-2, TBP-2 also affects autophagy. Previous studies reported that upregulation of TBP-2 mediates dysfunction of autophagy in hyperglycemia [38]. Meanwhile, TBP-2 promotes stress-induced autophagy via a REDD1/TXNIP complex [39]. Consistent with the previous findings, our study showed overexpression of TBP-2 in LECs which are more vulnerable to oxidative stress and triggered stronger autophagic response. Knockdown of TBP-2 partially reversed the effect, with milder autophagic response and moderate cell viability loss. Our research focused on the autophagic regulatory effects of TBP-2 under oxidative stress in lens.

With the help of autophagy inhibitors, we have demonstrated that TBP-2 mostly regulates the initiation stage of Akt activated autophagy and induces a higher level of autophagic flux. This result can be partially explained by the role of TBP-2 as a negative regulator of antioxidant system. Excessive ROS triggers reactive response including autophagy.

### 4.3. The Role of TBP-2 in Regulating the Upstream Pathways of Autophagy

We found that TBP-2 functions primarily in the initiation stage of autophagy; we then studied the regulating roles of TBP-2 in the upstream pathways of autophagy. The PI3K/Akt/mTOR pathway has a central role in autophagy regulation [40], and it also affects TBP-2 expression level [41]. In our study, we found that the regulation of TBP-2 on LECs under oxidative stress was through an mTOR-independent pathway and TBP-2 inhibited the bioactivity of Akt through dephosphorylation of Akt. Wortmannin also suppressed the autophagic flux regulated by overexpressed TBP-2, suggesting the intact interaction between TBP-2 and Akt. The mechanism of how TBP-2 regulates autophagy upon oxidative stress via Akt since the mTOR pathway is not involved requires further investigations.

**Figure 8:**
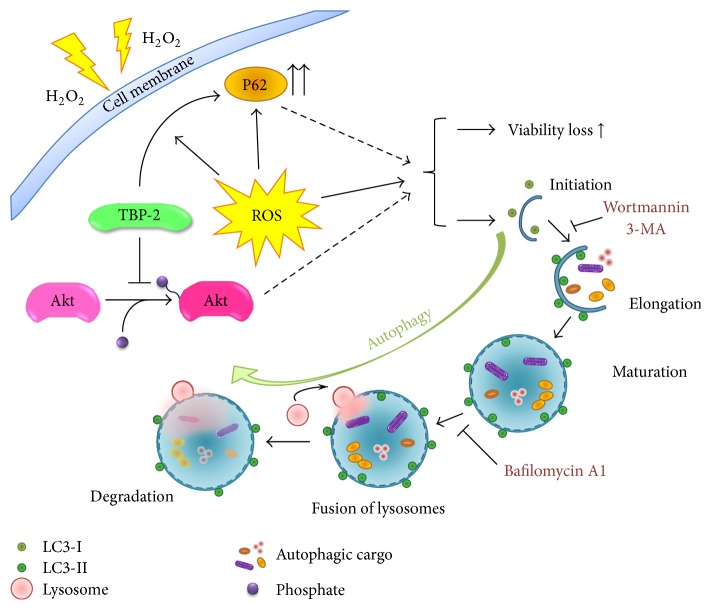
Potential mechanism of TBP-2's function in autophagy under oxidative stress. ROS, reactive oxygen species.

In summary, our findings suggest a regulating role of TBP-2 in human lens epithelial cells under oxidative stress. Oxidative stress can cause cell injury and autophagy in LECs, and TBP-2 promotes this response. TBP-2 mainly functions in the initiation of autophagy. It is mTOR-independent and might be caused by the inhibition of Akt phosphorylation under oxidative stress. A diagram of our findings and hypothesis is shown in Figure 8. Overall, our study contributes to our understanding of the functional characteristics of TBP-2 and its possible role in the development of lens cell injury and cataract. It may also allow the development of a gene screening approach that involves TBP-2 as a predictor of early-onset age-related cataract.
